# Recovered memories of trauma as a special (or not so special) form of involuntary autobiographical memories

**DOI:** 10.3389/fpsyg.2023.1268757

**Published:** 2023-12-07

**Authors:** Olivier Dodier, Krystian Barzykowski, Céline Souchay

**Affiliations:** ^1^APSY-V Laboratory, Department of Psychology, Literature, Languages and History, University of Nîmes, Nîmes, France; ^2^Applied Memory Research Laboratory, Faculty of Philosophy, Institute of Psychology, Jagiellonian University, Kraków, Poland; ^3^Laboratoire de Psychologie et Neurocognition, LPNC CNRS 5105, Université Grenoble Alpes, Grenoble, France

**Keywords:** recovered memory, involuntary autobiographical memory, false memory, traumatic memory, trauma, retrieval cue

## Abstract

Recovered memories of trauma are memories of traumatic events experienced generally during childhood, but of which the persons were unaware until they retrieved it. Legal decisions are sometimes based on such recovered memories, the validity of which is often questioned. Yet, people can recover genuine traumatic memories of childhood abuse. In this paper, we present and further discuss the idea that recovered traumatic memories can be interpreted in the context of the autobiographical memory framework. Specifically, we argue that recovered memories may be accessed after exposure to incidental cues that initiate unexpected spontaneous memory retrieval. Thus, we relate the recovered memory phenomenon to involuntary autobiographical memories and argue that it is an example of highly stressful, emotionally negative, and intense involuntary memories that were yet never recalled. This novel, evidence-based perspective leads us to reconsider the examination of the validity of eyewitness testimony as a continuum ranging from the least valid form (i.e., memories recovered in highly suggestive context facilitating its factitious reconstruction) to the most valid form (i.e., memories that were triggered by cues without any person’s voluntary engagement), and this in relation with how internal (e.g., age and internal cue) or external (e.g., suggestion in therapy, suggestion during investigative interview, and contextual cue) factors may influence memory retrieval. Finally, we propose several recommendations for experts that may be useful in assessing the validity of a testimony based on recovered memories.

## 1 Introduction

In this paper, we present and further discuss the idea that memory processes can contribute to the explanation of (at least) some instances of the phenomenon of recovered memories of traumatic events. This type of memories was the subject of heated debate during the “memory wars” in the 1990s (Crews, 1995). On the one hand, therapists considered that it was possible for traumatic memories to be repressed and thus pushed outside the boundaries of consciousness, before returning in their original form, notably through therapeutic methods (e.g., Bass and Davis, 1988). On the other hand, skeptics, mainly experimental psychologists, but also several clinical psychologists and psychiatrists, considered that such recovered memories were probably false memories suggested by third parties, generally psychotherapists (in this case memories of events that never took place, thus rejecting the hypothesis of traumatic repression, Loftus, 1993; Holmes, 1994; Lilienfeld and Loftus, 1999). The “memory wars” controversy came to a head when it reached the courtroom, where people were accused of committing sexual abuse based on memories recovered in psychotherapy (Loftus, 1993). This led to quarrels between experts, with supporters of repression on a side and skeptics explaining these accusations by induced false memories on the other. These disputes unfortunately turned an initially scientific debate into a popular one (see detailed examples in Loftus, 1993; see also examples of false memories allegedly induced by therapists in Kaplan and Manicavasagar, 2001).

Recent work has shown that memory wars still rage on (Dodier, 2019; Otgaar et al., 2019; Battista et al., 2023), with similar opposing camps. However, it appears that the debates have shifted towards neuroscientific approaches, with some authors (e.g., Markowitsch and Staniloiu, 2013; Chechko et al., 2018; Dimitrova et al., 2021) suggesting the existence of brain biomarkers of dissociative amnesia (another way of calling repression, Otgaar et al., 2019, 2023), while others have criticized the validity and lack of homogeneity of the results found in studies using brain imaging (Otgaar et al., in press). Closely associated with the topic addressed in our article, the debate has also shifted somewhat to something other than a repressed vs. false memory dichotomy. Specifically, while repression (or dissociative amnesia) is still criticized for its validity (Otgaar et al., 2019; Patihis et al., 2019; Battista et al., 2023; Pope et al., 2023), other explanations besides false memories are being put forth to explain recovered memories of trauma that may occur outside of a therapeutic setting. For example, some memories may not be fully encoded, due for example to stress that may limit the integration of certain information (Deffenbacher et al., 2004), or the use of substances such as alcohol or drugs (see Kloft et al., 2021; Segura et al., 2021). Another example is that in some cases the recovered memories of trauma are in fact continuous memories (i.e., memories of events that people feel they have always known occurred) that have only been reinterpreted as abuse with the time and maturity to understand the event (McNally and Geraerts, 2009; McNally, 2023). In this case, the event would be experienced in a non-traumatic way during encoding (because children are too young to understand the event, especially when it is of a sexual nature), with time the individuals do not think about it, before exposure to a contextual cue allows for the involuntary retrieval of an autobiographical memory, which can then take on its traumatic nature.

Specifically, our aim is to argue that recovered memories of actual traumatic events (i.e., true memories, as opposed to false memories, in this case memories of events that did not take place) are usually recovered after exposure to incidental cues that initiate unexpected spontaneous memory retrieval. In this context, after (i) a brief review of the literature on recovered memories of trauma, we will (ii) relate this phenomenon to the literature on involuntary autobiographical memories and argue that, at least in some cases, it may be an example of highly stressful, emotionally intense, and extremely negative involuntary memories that were yet never recalled prior to the unexpected memory recovery. Next, we will then (iii) propose that the validity of eyewitness testimonies (focusing on ones that come from recovered memories of trauma) may lie along a continuum ranging from the least valid memories (i.e., memories recovered in highly suggestive context facilitating its factitious reconstruction, e.g., during therapy) to the most valid memories that were triggered by cues without any person’s voluntary engagement (e.g., involuntary autobiographical memories retrieved unexpectedly during watching a movie). Finally, we will (iv) propose brief recommendations for expert witnesses that may be useful in assessing the validity of a testimony based on recovered memories of trauma.

## 2 Recovered memories of trauma

Recovered memories of trauma are memories of a generally stressful and distressing event that a person has, of which he or she was unaware until he or she remembered it (Ost, 2006). For example, in a case described by Dodier et al. (2023), a 16-year-old girl suddenly recovered her memory of sexual abuse by her great uncle when she was 8, after hearing his name in the middle of a discussion. In an interview, she said she did not know the abuse had happened until she retrieved it. Such memories are usually accompanied by a sense of surprise at (re)discovering the facts (Geraerts et al., 2007), to the point where they could lead to significant psychological distress upon recovery (McNally and Geraerts, 2009). Recovered memories, when they are traumatic in nature, can join the reversible feature of dissociative amnesia, as defined by the DSM-5-TR (American Psychiatric Association, 2022).

Traditionally, recovered memories of trauma have been the subject of debate between scientists and clinicians in the fields of research, therapy, and justice, as the validity of such memories is difficult to establish and therefore so is the examination of the likelihood of those memories being false. Indeed, much laboratory work, widely replicated, has highlighted the ease of creating false memories of entire events in people (Scoboria et al., 2017), including criminal events (Shaw and Porter, 2015; but see Wade et al., 2018). These criticisms were made because many cases of recovered memories of trauma occurred in a therapeutic setting, by some clinicians who were convinced that childhood traumas had been repressed, and that it was necessary to recover them in order to heal people. The problem was that the methods dedicated to recovering memories were highly suggestive and resembled in many ways the experimental methods used to access for creating false memories (Ost, 2006). While there is a clear difference between laboratory experiments and false memories in real life (which are usually traumatic), it is important to note that documented cases of suggested false traumatic memories have been reported in the literature (Loftus, 1993; Kaplan and Manicavasagar, 2001; Otgaar et al., 2022).

Clearly, not all recovered memories of trauma are false memories; we know of no memory specialist who would consider every recovered memory of trauma to be necessarily a false memory. Rather, our position is that it depends on the context in which these recovered memories are, in fact, recovered. This is consistent with our central idea that the validity of testimonies should be examined on a continuum, rather than in a purely categorical fashion.

As just mentioned, recovered memories of trauma are traditionally associated with the therapeutic context, to the point where there is a popular belief that it is necessary to recover repressed memories in order to heal various disorders that are believed to be unconscious expressions of childhood trauma (Otgaar et al., 2021). However, it appears that the vast majority of recovered memories of trauma occur outside of any therapeutic context, and even that individuals are alone when they recover the memory (Dodier and Patihis, 2021). The aforementioned study did point out that such recovered memories alone could result from suggestions (e.g., following discussions or self-documentation on the topic of repressed traumatic memories), but it also raises the question of the possibility that memories of events with traumatic potential can be recovered following exposure to a contextually derived retrieval cue, consistent with Tulving’s classic work on the specificity of encoding (e.g., Tulving and Thomson, 1973) and the multiple pathways to access a memory trace (Tulving, 1974).

This is related to the hypothesis put forward by McNally and Geraerts (2009) according to which recovered memories of childhood sexual abuse that actually occurred reflect that the individuals were too young at the time of the event to understand. The memory would then be encoded as a bizarre, confusing and unusual event (Clancy and McNally, 2005/2006), but not as traumatic. Thus, as demonstrated by experimental work on the forgetting curve (Murre and Dros, 2015), the strength of the memory trace of the event would decline. After a period of time that can vary from months to decades, a retrieval cue (e.g., hearing about the perpetrator, returning to the scene) would allow the involuntary and spontaneous retrieval of the memory, which would then be reinterpreted as sexual abuse. This hypothesis was corroborated by Dodier and Patihis (2021) showing that a third of the people claiming to have recovered memories of sexual abuse during childhood specified that they had never really forgotten it, but had reinterpreted it over time.

These different research findings then allow us to consider a new evidence-based approach to explaining recovered memories of trauma. More specifically, in the next section we will develop the encoding and retrieval mechanisms behind involuntary autobiographical memories, and how this work offers a powerful explanatory framework to account for such recovered memories.

## 3 Involuntary autobiographical memories as a possible framework to understand recovered memories of trauma

Our main idea put forward here is that, under certain circumstances, a memory of a traumatic/unpleasant past episode may simply pop into our mind without any preceding intention (i.e., when one did not try to recall a given memory). Importantly, such involuntary retrieval of a past episode may become a core element (or starting point) of a recovered memory of trauma (e.g., when one spontaneously experiences a past memory of being abused by a given person may start voluntarily thinking about that situation elaborating further on that experience giving a rise to recovered memory of trauma). While there may be several possible mechanisms of recovered memories of trauma (e.g., simple processes such as failure to remember a prior recall of the event and/or forgetting mechanisms; Otgaar et al., 2019), which may not be mutually exclusive, we focus on some instances of recovered memories of trauma as a result of involuntary autobiographical memory retrieval. To this end, we briefly introduce the self-memory system first (Conway and Pleydell-Pearce, 2000), followed by conceptualization of memory retrieval stages (Barzykowski and Mazzoni, 2022; Barzykowski and Moulin, 2023). Then, we discuss possible circumstances under which such recovered memories may be most likely to occur and, as we argue in the present paper, may be even a more valid representation of the event.

### 3.1 The self-memory system

The ability to remember our personal past; namely, anything that we have witnessed and/or experienced while being self-reflectively aware that a given remembered event belongs to our personal past is called autobiographical memory (e.g., Tulving, 1985; Conway and Pleydell-Pearce, 2000). Recent theories of autobiographical memory acknowledge two broad ways in which such memories can be accessed and which are the result of the presence or the lack of conscious intention (i.e., wanting to recall a given memory): involuntary and voluntary retrieval (Roberts et al., 1994; Berntsen, 1996; Schlagman and Kvavilashvili, 2008; for similarities and differences between involuntary and voluntary memories see also, Barzykowski and Staugaard, 2016, 2018; Barzykowski et al., 2019a). Therefore, each time we want to recall, for instance, a childhood summer holiday at our grandparents with more or less detailed events (e.g., *eating cherries directly from the tree*, etc.), we use our voluntary memories. However, sometimes such memories may come to our mind without any conscious attempt at retrieval, for example, when watching a movie, a memory of *having delicious cherries with grandparents during childhood holiday* may simply enter our mind without being sought-for. While involuntary autobiographical memories were somewhat ignored for several decades (e.g., Miller, 1962/1974), they are now considered as a basic mode of remembering, central to psychological well-being and, importantly, frequently experienced in a daily life (e.g., Berntsen, 2010; Rasmussen and Berntsen, 2011; Uzer et al., 2012). An important result from a naturalistic diary study, among others, is that involuntary memories arise in response to incidental (both internal or external) cues that usually overlap with key features of the memory content (Berntsen, 1998; Schlagman et al., 2007; e.g., *seeing a cherry may trigger a certain past episode of picking and eating them in grandparents’ garden*).

According to the influential model proposed by Conway and Pleydell-Pearce (2000; for later modifications see also: Conway, 2008, 2009; Conway and Jobson, 2012), autobiographical memory consists of a hierarchical network of interconnected nodes that differ in terms of their level of specificity. At the top of the network are superordinate levels of important periods of one’s life (e.g., *when being a child*), general events (e.g., *holiday*) and common themes (e.g., *summer holiday with grandparents*). At the bottom of the network are stored fragments of events with specific sensory details (e.g., *details experienced when picking/eating cherries directly from the tree*). Higher levels are constituted by such basic and specific memory contents. Importantly, the self-memory system and the ability to remember personal past emerges over the years of cognitive development (e.g., language). For instance, there is a strong relationship between language development and memory showing that the better language skills, the better (more efficiently) autobiographical memory works (e.g., Fivush and Hamond, 1990; Leichtman and Ceci, 1993; Nelson, 1993). This means that over the course of cognitive and language development children are better at taking control over their memory, being able to more efficiently: encode events (knowing what is important, paying attention to the events, elaborating them to be better remembered, understanding an event within a broader context), store events (as they are stored in relatively stable, organized cognitive schemas, scripts and knowledge nodes/units), retrieve (as they are better at using cues and strategies to recall a given event).

### 3.2 Conceptualization of memory retrieval stages

**Figure 1:**
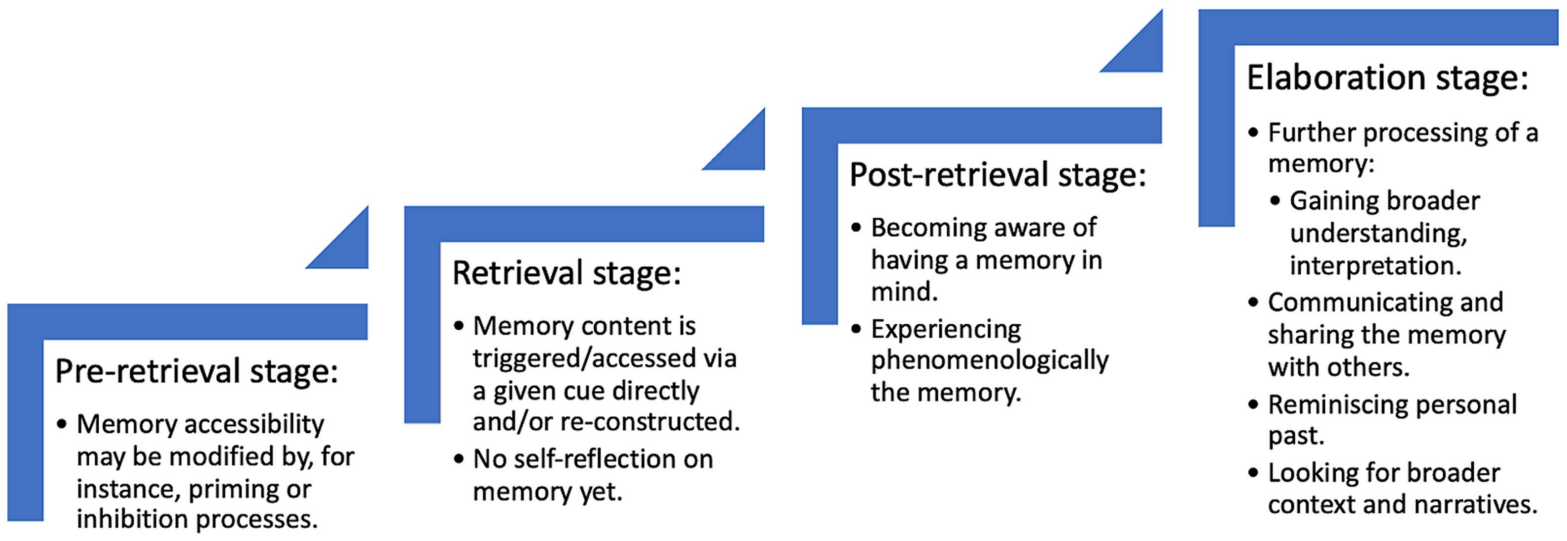
The theoretical and demonstrative conceptualization of autobiographical memory retrieval to better disentangle the possible factors operating during a memory retrieval as developed and described by Barzykowski and Mazzoni, 2022 and Barzykowski and Moulin (2023). These stages are thought to be as a dynamic system, where the retrieval process can jump back and forth and even activate several stages at any given time. A helpful analogy is a neural network.

Conway and Pleydell-Pearce (2000) suggested that retrieval of a given memory is due to the activation of autobiographical information that spreads across the network. Furthermore, such activation may be elicited by different types of cues leading to either generative or direct retrieval. While generative retrieval is a result of a top-down cognitively controlled search process (i.e., we know what memory we want to recall and we do our best to do so), direct retrieval is thought of circumventing the search process accessing a memory very quickly (and thus is beyond our control as it happens to us rather than we have control over it). Conway and Pleydell-Pearce (2000) argued that fragments of memory representations are constantly activated at the bottom level of the hierarchy by different internal and external cues—i.e., those in the environment, those acted upon by the recognition memory system; but the vast majority of such memories cued by internal and external cues never reach consciousness. While there are several hypotheses of why such memories do not reach the awareness threshold (see for instance Vannucci et al., 2015; Barzykowski et al., 2019b), from the perspective of the present paper it is more important to reflect on the question of how memories are successfully retrieved. As presented in Figure 1, to simplify the act of memory retrieval, it may be conceptualized into four following demonstrative stages (Barzykowski and Mazzoni, 2022; Barzykowski and Moulin, 2023): (1) pre-retrieval stage (e.g., modifying memory accessibility by priming), (2) retrieval stage (forming and developing a memory), (3) post-retrieval stage (becoming aware of a memory and further its processing), and (4) retrieval outcome report stage (giving verbal account and gaining understanding of a memory and past episode) (see Figure 1).

The pre-retrieval stage (1) may be associated with any cognitive processes that either facilitate or impair retrieval. More precisely, during the pre-retrieval stage an individual may be in “*retrieval mode*” in which “*the cognitive system is prepared for or expects memory construction and recollection*” (Conway, 2001, p. 1379). For instance, one may be occasionally (i.e., incidentally and peripherally) exposed to some information more or less directly relating to childhood abuse. This may lead to the effect of priming which, for some memories, may enhance the likelihood that a given memory will be triggered and/or will enter a person’s awareness (e.g., seeing children).

The retrieval stage (2) relates to the forming and development of an autobiographical memory. According to the self-memory system, any memory information stored in the autobiographical memory system may be: (a) retrieved automatically without any conscious intention, and/or (b) triggered by internal or external cue, and/or (c) activated by spreading activation mechanism. This means that while we may have an access to memory content we know (or think) exists in our memory (but there are some exceptions, e.g., non-believed memories, see Mazzoni et al., 2010), there may also be instances of retrieval of memories that were either (i) forgotten (inaccessible and/or unavailable but recognized as known and experienced in personal past, e.g., *a memory of cherry picking in my grandparents’ garden popped in my mind, I forgot about it but now I remember it*) leading to strong feeling of surprise (as in the proustian-like memories) or (ii) rediscovered (inaccessible and unavailable and, importantly, recognized as not known before as experienced in the personal past, e.g., *I have just remembered a neighbor touching me up when I was picking cherries in my grandparents’ garden*). In general, during the retrieval stage (2) a memory is triggered by and/or accessed via a given cue, and it may be either reconstructed, directly retrieved, voluntarily searched or involuntarily recalled, depending on the memory pre-retrieval and retrieval processes involved. It is also worth underlining that such a memory retrieval may be without explicit self-reflection; namely, a given memory might have been formed but one may not be explicitly aware of it yet (something that refers to an “*experiential level of consciousness*”; Baird et al., 2013).

Once the memory is formed, during the post-retrieval phase (3), people may become aware of having the memory in mind. Thus, this stage relates to the ability to, for example, extract autobiographical content from the stream of consciousness to explicitly become aware of having a memory that is autobiographical (this is the level of meta-awareness). At this stage, one is fully aware that an autobiographical memory was actually retrieved but no further understanding or interpretation is done yet. Importantly, becoming aware of an involuntary memory popping into mind is most frequent when one is engaged in an attentionally undemanding activity (e.g., driving a car, washing a dish etc., for experimental studies see Vannucci et al., 2015; Barzykowski and Niedźwieńska, 2018a).

In the last stage (4), the retrieved memory may be shared with others and reported by giving a verbal account of the content. This is a stage during which a broader understanding of a given memory is developed. Depending on the context of that memory (e.g., *memory of sitting on a Santa-Claus laps while having a photo in a shopping mall* vs. *sitting on a lap of a given person while being alone in a bedroom*), one may come to a conclusion that a given memory may be understood as an experience of, for instance, childhood abuse, although it was not encoded/understood in such a manner at the time of the event occurrence. Furthermore, different events may be encoded/remembered with various levels of emotional intensity. As demonstrated in previous studies (e.g., Barzykowski and Staugaard, 2016, 2018; also, studies showing that emotional memories contain more details, e.g., D'Argembeau et al., 2003; Comblain et al., 2005) the general rule is that the more emotionally intense an original event was, the easier a given memory should pass the awareness threshold making ones’ self-aware of having a memory of that event (Stage 3). However, in some cases, especially if the event was not fully understood, emotions may arise in response to interpretation of an event made in Stage 4 when a broader understanding of it is gained. Put differently, a child may not fully understand the event of abuse. However, once it is recalled as an adult, the event may be reinterpreted as such given the knowledge one has about what may or may not be defined as an abuse.

### 3.3 Recovered memories of trauma as a form of involuntary autobiographical memory

We argue that recovered memory of trauma relate to a broader concept than involuntary memory retrieval; namely, while typical memory of a past event is experienced and realized by a rememberer (i.e., one may recall sitting on someone’s lap in a bedroom), an involuntary memory may become a recovered memory of trauma when it is interpreted and understood as an abuse. However, there are two questions to be asked; namely, (1) why an event was not remembered earlier so a person is surprised when it is involuntarily recalled for a first time and (2) why and how such a memory is eventually recalled at a given time? These questions relate to two broad threads of encoding (the first thread) and retrieval (the second thread). We briefly elaborate further on these two threads.

The first question regards the reasons why a given event was not remembered earlier so one might not have been aware of experiencing an event in the past until it was spontaneously recovered. For instance, it may be argued that the recovered experience at the time of occurrence was not easily and straightforwardly understood by a rememberer. This challenges the efficient coding and of the memory, making it more prone to be forgotten (if it was indeed remembered in the first place) and/or rendered unavailable (i.e., difficult to access). Thus, a given incident may not be fully processed and encoded within an autobiographical memory base and may therefore be less accessible via a top-down (generative) memory retrieval process. This makes such a memory difficult to be accessed as, in general, the better an event fits an already existing cognitive schema/script, the easier and better it is later remembered (e.g., Pillemer et al., 1994). This was also suggested by McNally and Geraerts (2009); namely, that some instances of childhood abuse may be experienced and encoded as confusing and bizarre, but may not be traumatic. Also, the less frequent the recovered experience is, the lower the likelihood is that the memory about that experience will be accessible and rehearsed. Repeated events of past abuse that follow the same/similar pattern may be better encoded and remembered even if not fully understood (but such an understanding of the situation may be reached over time). At the same time, (non-traumatic) events that happened once may be relatively easily forgotten (i.e., when not having the possibility to rehearse and elaborate on the event). Put differently, it may be relatively likely that a recovered memory of trauma will relate to a single event that might have not been sufficiently encoded (e.g., processed, elaborated, understood), and therefore remains unavailable for a controlled and voluntary retrieval. Yet, a memory could be still retrieved if only automatic processing of cues in the environment detected some conceptual or perceptual overlap with stored memory representations of such long-forgotten event. Therefore, one would not only expect involuntary memories to arise (as we observe in an every-day life) but some of these memories on relatively rare occasions may relate to the personal past that one was not fully aware of (which is also observed in an every-day life). For instance, one may not remember meeting a given person during the conference but then seeing that person may trigger that forgotten memory.

The second question relates to the reasons why a given memory was recently retrieved. A critical issue we develop here is the cue-dependency of involuntary memory retrieval, that is, involuntary memories may be triggered spontaneously by any type of cue even when the rememberer does not expect a given memory to be retrieved. For instance, there may be a higher likelihood of recovering a memory about some past experience if such experience was combined with some attention-catching, unique or focal cue. This is not to say that one has to be self-aware of that stimuli but that that stimuli may be encoded as a somewhat vivid element of that event. The more unique and distinctive the accompanying cue is, the higher the likelihood is that such cue will efficiently trigger a given memory (see for instance Berntsen et al., 2013). As mentioned above (and as suggested by Barzykowski and Moulin, 2023), as our cognitive system automatically matches, as quickly and effortlessly as possible, the contents of mental representations stored in memory with the current contents of perception/attention, a spontaneous retrieval of a past memory is more likely to occur, if the memory contains something unique, distinctive that may trigger that representation. For instance, a unique scent accompanying a given event that might have been encoded/memorized alongside, may thereafter trigger such memory when one is exposed to this scent again. Conversely, if there are no distinctive cues encoded with an event, it may decrease the likelihood of incidental involuntary retrieval. We elaborate on the issue of cues below.

The uniqueness of a cue is somewhat crucial as according to the principle of cue overload, it is most likely that a cue will match several past events. Berntsen (2009) proposed a mechanism of cue-item discriminability, defined as “*how easily a given cue isolates an item*” (Rubin, 1995, p. 151 as cited in Berntsen, 2009, p. 107). That is, the more events that are associated with a particular cue, the less efficient this cue will be in triggering any one of them. This was also demonstrated by Berntsen et al. (2013); namely, that involuntary episodic memories are retrieved more often in response to unique compared to repeated (i.e., associated with many memories) cues, which confirmed the principle of the cue-overload. Therefore, the more unique a cue accompanying the recovered experience is, the higher the likelihood is that such a cue may trigger abruptly and involuntarily a memory of that experience. This does not imply that a less unique cue cannot trigger a recovered memory of trauma. This is particularly evident since any type of cue has the potential to elicit a memory of that event, if only it was present during the recovered experience. For instance, priming processes (i.e., increasing the activation of a memory information by prior encounter with the contents of memory representation) may actually increase such a cue-item discriminability allowing for efficient activation of a particular involuntary memory (e.g., Mace, 2005; Barzykowski and Niedźwieńska, 2018b). It is also possible that on some occasions an environment/surrounding setting may consist of cues that map onto a given past memory or there is a relevant configural, contextual similarity between the current situation and a given past events. While these cues/contexts may not be efficient in triggering a given memory, it is nonetheless possible that some of them may increase the accessibility of that memory. In other words, over time, recurrent exposure to cues might lead to the memory being fully retrieved.

### 3.4 Conclusion

The idea that recovered memories of trauma may be a form of involuntary autobiographical memory suggests that they may reflect quite authentic events and therefore be very valid. This raises the question of the context of recovery, and more precisely of the variations in the different contexts of retrieval of recovered memories of trauma. Insofar as these may vary, then the validity of the memories may vary because these contexts may be more or less suggestive. Thus, we explore in the following section how the validity of memory reports can be examined in a legal framework. We propose that maintaining an opposition between false and true memories, or between valid and invalid testimonies might be counterproductive. We therefore present in the following section that memory reports in a legal framework should be examined on a continuum of validity, rather than in a category-based approach.

## 4 Testimony validity as a continuum

Recovered memories of trauma may reflect events that never happened, as well as memories of perfectly genuine events (McNally and Geraerts, 2009). While these two phenomena may be labeled as, respectively, “false” and “true” memories, we propose that the validity of eyewitness testimony should not be viewed solely through the lens of “true” vs. “false memories.” The accuracy of a witness’s report can be considered in a more balanced way, since, for example, the term false memory is more “a linguistic convenience” than a truly unified phenomenon (Bernstein et al., 2018, p. 161), and can in fact refer as much to an event that never happened, as to elements of an event that actually did happen (in which case, the quantity of false elements can widely vary from one individual to another, in the same individual, and from one event to another).

Another (while related) argument is that there is now a vast body of literature showing that there is no relationship between different forms of false memory. Specifically, sensitivity to one type of false memory (e.g., spontaneous false memories, elicited in particular through the DRM task; Deese, 1959; Roediger and McDermott, 1995; see below for more details) does not predict sensitivity to other types of false memory (e.g., misinformation effect, Loftus, 2005; Loftus and Klemfuss, 2023; or creation of rich false memories, via the lost-in-the-mall paradigm, Loftus and Pickrell, 1995). Bernstein et al. (2018) therefore suggested that different constructs may underlie the different types of false memories. Mazzoni (2002) proposed that false memories could be distinguished by their origin: suggestion-dependent false memories (i.e., misinformation effect, rich false memory creation), and false memories produced by the reconstructive nature of memory (e.g., DRM paradigm, schema-based false memories).

**Figure 2:**
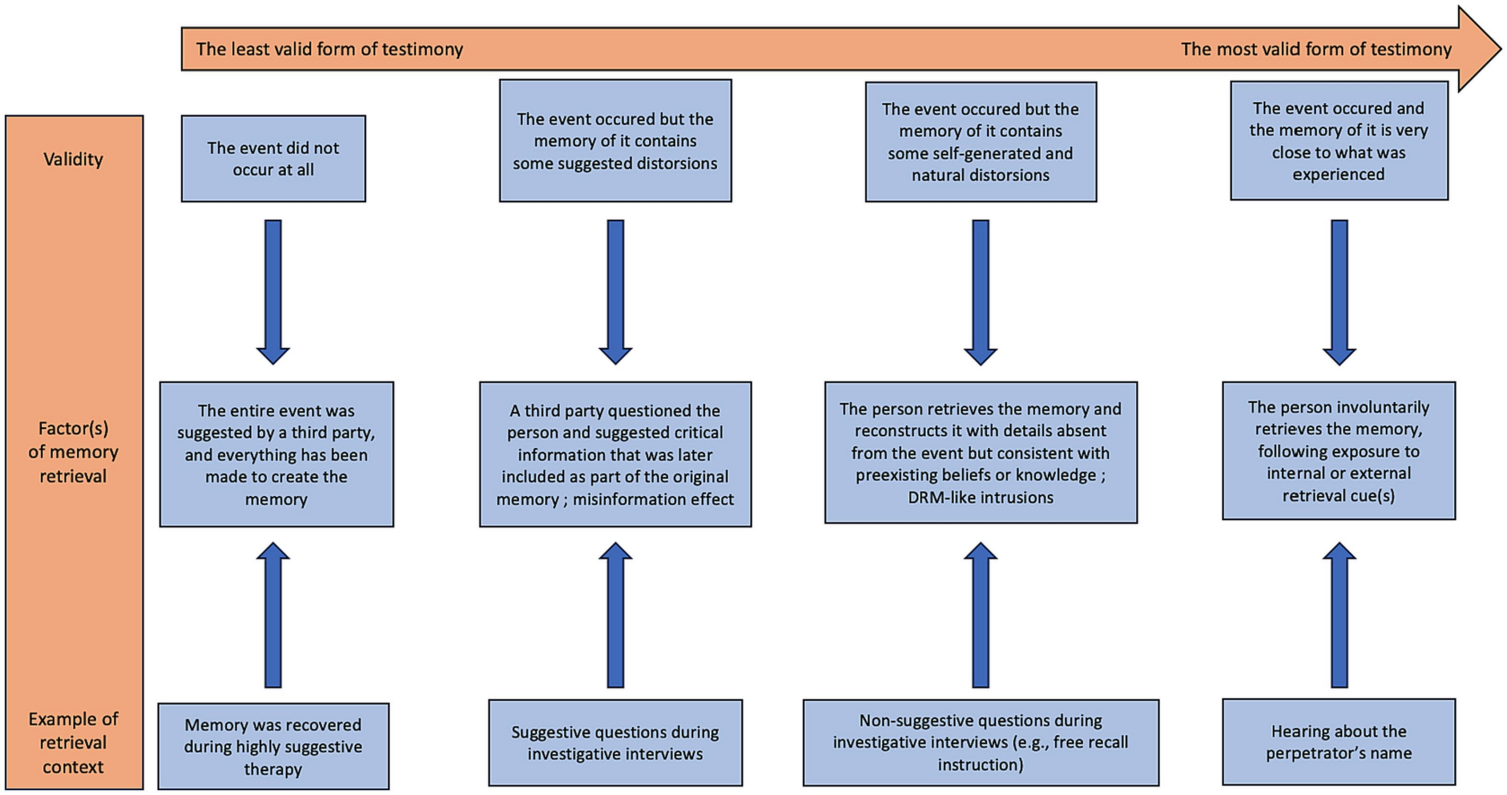
Continuum of the validity of memory reports in legal contexts.

What can be drawn from those arguments is that there might be a variety of memories different in their nature and validity. As a result an eyewitness may be based on a recovered memory of trauma, which may reflect an event that never happened or an event that actually did happen. It may also be a continuous memory and have been more or less distorted by external suggestions and/or a natural (and internal) reconstruction process. We therefore believe that expert witnesses (e.g., forensic psychologists, memory experts, clinical psychologists) should assess the validity of eyewitness reports as a continuum, ranging from the least valid form (i.e., recovered memory of a traumatic event that never occurred) to the most valid form (i.e., involuntary autobiographical recovered memory of a traumatic event that did occurred) (see Figure 2).

### 4.1 The event did not occur at all

We consider that the least valid form of testimony based on a recovered memory of trauma is when the event did not occur at all. Typically, this type of memory is the result of suggestion by a third party, where several techniques have been used to recover (and, in this case, create) the memory. In the laboratory, the seminal study to highlight the possibility of individuals developing memories of events that never occurred was that of Loftus and Pickrell (1995), traditionally known as the “lost-in-the-mall” study. In this study, the experimenters provided participants with a booklet containing three true autobiographical accounts of their childhood, as well as a false autobiographical account according to which the participant, then a young child, had become lost in a shopping mall. This false event was constructed to be credible (i.e., including true biographical elements). On the basis of this booklet, participants were then asked to indicate what they remembered about each of the events, all presented as true, with the option of specifying that they had no recollection. Logically, in the first interview with the experimenter, the false event was given very little detail. Participants were then interviewed twice more, between which they were invited to think about the events, try to remember them and imagine what might have happened. In the experiment, at the last interview, around 25% of participants partially or fully remembered the false event.

While this study has been criticized for its lack of ecological validity (e.g., the events in these studies do not equate with a traumatic event, Pezdek et al., 2006), and statistical issues (Crook and McEwen, 2019), there is evidence to support the conclusion that it is possible to induce detailed memories of events that never happened. Firstly, a very recent preregistered replication, with a larger number (*N* = 123) of participants than in the original study (*N* = 24), showed that 35% of participants recalled getting lost in a shopping mall during childhood, even though this event never occurred (Murphy et al., 2023). Secondly, a meta-analysis (i.e., raw data from several studies are aggregated for a broader analysis) showed that, in studies using this paradigm, around 30% of people came to remember and detail an event they had never experienced (Scoboria et al., 2017).

Also, a number of studies showed that highly suggestive methods could also lead people to recall such events that had never occurred, such as guided imagery (Hyman and Pentland, 1996), dream interpretation (Mazzoni et al., 1999), or hypnosis (Green et al., 1998). Interestingly, it appears that memories recovered in therapy are frequently the result of such methods (Dodier et al., 2019; Patihis and Pendergrast, 2019).

We consider this to be the least valid form of testimony, insofar as everything has been done to create a recovered memory of trauma without knowing or not if the experience of the event actually happened. Thus, by thinking of giving retrieval cues (or by deliberately trying to induce a false memory, see example below), an individual could then suggest a whole scenario to a person that they could endorse, even if it does not correspond to any personally experienced event. In the worst-case scenario, the event simply never happened, and the risk of a miscarriage of justice is greatly increased if no one in the judicial process properly assesses the validity of the testimony.

For example, in 2012, a French psychologist was convicted of moral abuse for deliberately suggesting memories of events that never actually occurred in two patients. It was established that these events had never been experienced by the two victims, as they were intrauterine memories of attempted abortions. By inducing the victims to develop such memories, the psychologist led them to break their family ties and make them dependent on his therapeutic care, guaranteeing him substantial income (for more information, see: https://www.lemonde.fr/societe/article/2012/04/13/le-proces-d-un-therapeute-accuse-d-inventer-de-faux-traumatismes-a-ses-patients_1684943_3224.html, article in French).

It should be noted, however, that these cases represent anecdotal evidence, and that recent work shows that cases of recovered memories in a therapeutic setting represent a minority of cases of recovered memories of traumatic events (Dodier and Patihis, 2021), and that this seems to be independent of the therapy type (Dodier et al., 2019; Patihis and Pendergrast, 2019). There is also anecdotal evidence that recovered memories of traumatic events during therapy may correspond to events that truly occurred (Schimmenti, 2017). This reinforces the need to conceive of the validity of testimonies as a continuum and to be able to explore the precise context in which memories are recovered to give an expert opinion.

### 4.2 The event occurred but the memory of it contains some suggested distortions

Progressing along the continuum, we arrive at memories relating to events that actually happened, but which contain details that never occurred in the event, and which were suggested by third parties. When people incorporate into their memory information suggested by others (or by the media) after the event has taken place, this refers to the misinformation effect. Decades of research have demonstrated the robustness of this effect and the extent to which memories are sensitive to external factors and therefore can be reconstructed by including encoded post-event information (Loftus, 2005; Loftus and Klemfuss, 2023). This effect can occur with very explicit suggestions, where a person directly suggests the information that then could be included in the memory (e.g., “so he was holding a knife, right?”). However, it was shown in early work that a simple variation in the violent connotation of a car accident (i.e., use of the conjugated verbs “collided,” “bumped,” “contacted,” “hit,” or “smashed” to illustrate a car accident) could significantly alter the proportion of people falsely recalling seeing broken glass in the accident, when there was none (Loftus and Palmer, 1974).

The misinformation effect can arise from multiple sources such as co-witnesses (see, e.g., Hope et al., 2008; Jack et al., 2014), relatives, or police investigators (Loftus, 2005). The latter case is particularly sensitive, as investigators generally have information (or even assumptions) about the course of events and, without training in questioning techniques, can easily come to suggest information (e.g., Launay and Py, 2015; Verkampt et al., 2021).

We consider these memories to be more valid than those in which the event did not occur, because in this case (this will sound trivial), the event did occur, but in a different form to that represented in memory. However, distorted aspects are relative to assumptions made by others, and can sometimes even be the result of confirmation bias, where investigators already have a precise idea of how the event took place, and seek to confirm it by directing their questions (concerning child sexual abuse, see Zhang et al., 2022), and the probability is therefore high that these assumptions are related to critical information that can redirect an investigation. Thus, even without malicious intent, the memory may adopt a form desired by a third party, and thus its validity is highly questionable.

Take, for example, a situation where a person gives evidence of a bank robbery. During the interview, the police officer asks the witness “Was the robber carrying a weapon? A gun?” Regardless of the immediate answer given by the witness, the misinformation effect would be that, during a second interview, the person spontaneously reports that the robber had a firearm, when they did not. In this case, the person would have included in their memory of the event, even though they had really experienced it, the presence of a firearm which did not exist in the event.

### 4.3 The event occurred but the memory of it contains some self-generated and natural distortions

Since Bartlett’s seminal work (Bartlett, 1932) showing how cognitive schemas can modify and adapt memories to bring them a certain coherence, it has been widely accepted that memory is reconstructive, and that in the absence of external suggestions, our memories are comprised of our personally lived experiences, but also of intrusions, often semantic. Work on the DRM paradigm (Deese, 1959; Roediger and McDermott, 1995), which has been widely replicated, confirms this natural aspect of reconstruction. In this paradigm, a list of words (e.g., dream, pillow, yawn, and tired, etc.) is presented and linked to a critical lure (e.g., sleep), which is absent from the list. Typically, during a recall or recognition task, the critical lure is frequently falsely recalled or misrecognized as part of the list.

These distortions correspond to the ordinary functioning of memory and have an adaptive nature, in the sense that they can enable memories to be enriched with relevant information, maintain a certain coherence and can make it easier to plan future events or solve problems (i.e., Schacter, 2012). Their causes are twofold: they result from the activation of knowledge networks at encoding, contributing to their more or less explicit encoding with the event, but also from difficulties in identifying the source of memory encoding (e.g., the event vs. one’s own thoughts) (Roediger et al., 2001). Since they result from activations of knowledge networks during encoding, these intrusions are often consistent with the event experienced. While in everyday life, such errors do not seem to pose major problems, the impact is different in the forensic setting, where details can sometimes be of great importance (e.g., remembering the presence of a weapon, when there was none).

Despite this, it appears that these memories can be considered more valid than the memories previously described in this section insofar as (i) they are perfectly natural and they seem to concern everyone, including people with exceptional memory and autobiographical abilities (Paihis et al., 2013), (ii) unlike memories induced by external sources, it is extremely difficult to be able to estimate which details may be distortions or not in the absence of corroborating or contradicting evidence. Moreover, such intrusions may well occur when police interviews are conducted in an entirely appropriate way, without suggestions and with free recall tasks that can increase self-generated retrieval cues of erroneous information (e.g., see work on the cognitive interview, where an increase in errors is generally observed with this tool compared to a control tool; Memon et al., 2010).

It should also be noted that this phenomenon is dependent on internal factors, such as age, a phenomenon known as developmental reversal, according to which children are less sensitive to this type of distortion than adults (and therefore make fewer recall or recognition errors in a DRM task), because their knowledge networks are less mature (Howe et al., 2011). In adults, we also generally observe an increase in these distortions with age (e.g., Colombel et al., 2016).

Consider the example in the previous section, where a witness describes the robbery that they experienced. Assume that the police officer does not suggest the presence of a firearm. Because the “gun” information is semantically consistent with the “robbery” event, it would be possible for the witness to generate and include this information in their memory of the event, which they would have truly experienced, without any external influence. In this case, their prior knowledge (e.g., script) could have led to the spontaneous intrusion of the “firearm” information into the “robbery” event. In the same way, the person could also have included other information semantically linked to the concept of “robbery,” such as the fact that the robber was wearing a mask, or that they said certain words such as “nobody moves,” etc.

In the case of natural distortions (or suggested distortions, see previous section), the validity of testimonies is greatly reduced because distortions can be very relevant to the investigation and thus redirect it completely (e.g., suggesting a physical characteristic of a suspect, when the perpetrator is someone else) and/or be very consistent with the event, and therefore credible, and potentially aggravate the situation (e.g., presence of a gun when there was none).

### 4.4 The event occurred and the memory of it is very similar to what was experienced

While there are several methods used to study involuntary autobiographical memories (e.g., semi-structuralized diary methods, questionnaires), a typical laboratory-based method (originally developed by Schlagman and Kvavilashvili, 2008) is built on the observation that involuntary memories are most frequently triggered by easily identifiable cues present in the closes surrounding (most of which are verbal-type, e.g., heard/read words). Thus, in this method participants are engaged in a monotonous vigilance task (detecting seldomly occurring pattern of vertical lines) while exposed to irrelevant word phrases (e.g., birthday party and upsetting conversation etc.) some of which may incidentally trigger involuntary memories. Importantly, participants are also instructed to write down any spontaneously occurring thought and/or memories during performance of the vigilance task. Such laboratory-based procedure provided in recent years experimental way to investigate involuntary memory retrieval under well-controlled conditions. As mentioned earlier, an interesting consequence of involuntary autobiographical memory retrieval, as described in the previous section, is that such memories are activated at the bottom level of the hierarchy by different internal and external cues circumventing the search process (also schemas and cognitive scripts), they may be considered as more valid. For instance, Barzykowski and colleagues (e.g., Barzykowski and Staugaard (2016, 2018; also, Barzykowski et al., 2019a) demonstrated in a well-controlled experimental condition that while some memories may require very little reconstruction and other memories rely more heavily on reconstructive efforts, reconstruction may not be required specifically for memories that are involuntarily retrieved. Therefore, we argue that involuntary autobiographical memories, following exposure to an internal or external retrieval cue, may be most likely to reflect the event as it occurred.

Put differently, it may be that a recovered memory of trauma retrieved in response to an external/internal cue directly relating to something presented during the occurrence of an original event may be the closest approximation (especially when compared to voluntary retrieval) of something that occurred in the past. For instance, Staugaard and Berntsen (2019) provided evidence demonstrating that indeed over time memories for past events become more cue-dependent which may eventually hamper strategic and voluntary retrieval at longer delays. Interestingly, they also showed a somewhat steeper slope of the forgetting curve in the voluntary compared to involuntary memory retrieval (Staugaard and Berntsen, 2019). When discussing their findings, they also suggest the plausible link between recovered memories and involuntary memories as follows (p. 903–904): “*The unexpected activation of dormant memories in response to situational cues is also consistent with some observations in clinical psychology of “recovered memories” of childhood trauma (see, e.g.,* Conway, 1997; Read and Lindsay, 1997, *for reviews). Although the notion of recovered memories is contentious, and although the majority of such recovered memories appear to have been brought about through strategic retrieval attempts in the course of psychotherapy* (*e.g.,* Geraerts et al., 2007)*, there are some examples of recovered memories outside of therapeutic settings in response to situational cues (e.g.,* Bendiksen, 1997*), which might be conceptualized as involuntary memories of forgotten events. However, the fact that the present studies used laboratory material without the personal significance and levels of complexity associated with real-life events renders these possibilities highly tentative and speculative*.” We fully agree with the authors on this idea, and we also call for more studies on the possible mechanisms underlying such resurfacing of the past events in every day context. As for now this issue and its implications for memory accuracy still requires a robust and thorough discussion. In all cases, we do not argue though that such a memory may never be distorted but that there may be a high likelihood that such memory actually happened, especially if it relates to an event with a distinctive and noticeable cue (see also below).

The presented idea that recovered memories of trauma based on involuntary autobiographical memories may be highly valid is based on the assumption that a cue serves as a way to access a memory information that already exists within the autobiographical memory base in a form that it was encoded rather than launches a reconstructive memory retrieval process. Thus, it is rather unlikely that a cue can readily access information that was not previously presented (encoded or experienced) within the memory system, at least under typical circumstances. However, if only previously suggested, imagined, elaborated, that is, in any way “artificially created,” a cue may also trigger such a memory representation that although existing in the memory system remains rather false.

For example, a person who spontaneously recalls, in detail and without any effort at retrieval, having been subjected to violence during their adolescence by their neighbor, on hearing his name during a discussion, could have a memory, at the time of retrieval, in the version closest to what they have encoded. For this reason, it is crucial for a testimony to be collected promptly and in an appropriate manner (i.e., free from suggestion and based on free recall) following the recovery of a memory in such a context. This helps minimize the risks of contamination or excessive reconstructions resulting from numerous retrieval efforts.

Thus, a proper analysis of the context of memory retrieval (e.g., unexpectedly retrieved for the very first time and not elaborated, developed in any way previously) and memory content *per se* (e.g., the correspondence between triggering cue and the memory content) may help in evaluating the memory’s validity. We elaborate on this idea in the last section of the article.

Finally, it is also important to highlight and explicitly stipulate that while describing the continuum, we refer to “*the most valid*” memory. However, we do not imply that this memory is 100% accurate or it is not susceptible to distortion, fading, false details or any other mechanism(s) underlying memory erroneous retrieval. We rather would like to argue that such memory may be, in some cases, the closest approximation (compared to voluntary retrieval) to the representation of the original event and the way it was initially encoded. While we present and further develop such an idea, we are fully aware that future studies and more empirical data are needed.

## 5 Brief recommendations for practice and concluding remarks

Although for reasons of clarity and practicality we may give the impression that we have identified four types of testimony, it is more appropriate to consider them as dimensions that can be placed on a continuum (see Figure 2). Each of these dimensions may overlap with others, and the aim is that expert witnesses assess how valid a testimony is, rather than identifying to which category it belongs. For example, a memory may have been induced in therapy, but concern only an entire part of an event that actually took place in the first place (e.g., a family meal that actually took place, but where the subsequent acts of sexual abuse are totally suggested by a therapist). It is also quite likely that a memory will include details suggested by an investigator, as well as self-generated and natural distortions. As a final example, a memory of childhood abuse may be recovered spontaneously following exposure to an environmental cue, but may then have been recalled repeatedly with suggestive questions by, for example, family members, friends, and/or investigators.

Most existing tools for discriminating between true and false testimonies have theoretical and practical limitations. For example, the *Criteria-Based Content Analysis* (Steller and Koehnken, 1989; see also Volbert and Steller, 2014 for an update), one of the four parts of the Statement Validity Analysis (Granhag et al., 2015) has focused most of its work on detecting lies and deception, based on the idea that an invented narrative will be qualitatively different from one based on a real memory. Limitations in the tool’s validity have been pointed out (Rassin, 2000). When applied to the distinction between true and false memory, it appears that this tool is of limited use (Kulkofsky, 2008; Volbert and Steller, 2014). Another example is the *Reality Monitoring* (RM) framework, which aims to distinguish between internally and externally generated memories (Johnson and Raye, 1981). However, the ambiguity of the definitions of what is internally generated and what is externally generated was highlighted very early on (Johnson et al., 1993). Similarly, the aim was rather to detect deception or lies. Here again, the usefulness of RM in distinguishing between true and false memories seems quite limited (Otgaar et al., 2010). In any case, such tools aimed at identifying markers of truthfulness in testimonies serve to categorize testimonies as true or false, and in our view represent a reductive view of the validity of testimonies.

The role of expert witnesses when evaluating eyewitness testimonies is therefore to be able to weigh the extent to which the event contains, for example, suggested information, in order to place the testimony on the overall dimension of validity. This requires a precise, rigorous and thorough assessment of the context in which a memory has emerged. The following questions can be asked when assessing the validity of a memory report: Is the memory retrieved? If so, how? As a result of discussions? If so, with whom? In what form? After how long a discussion? If not, spontaneously? Under what circumstances? What was the likely cue that activated the memory and led to its retrieval? Etc. If the testimony stems from a continuous memory (i.e., one that has not been suddenly recovered), has the person discussed it with others? If so, who? Were any questions put to the person during these discussions? Which questions? How did the investigators gather their testimony? How many times has the same event been recalled? Over what period of time? Etc.

It is necessary to explore all the elements in a case file in order to make a critical assessment of the retrieval contexts (i) of the memory in general, (ii) if possible, of all the critical information. In this way, expert witnesses will be able to better assess the validity of the testimony in detail, and not by relying on a binary conception of “true vs. false memory,” which may certainly simplify the understanding of triers of facts, but which probably reduces too much the form that memories can take, and the underlying mechanisms that enable them to be created. This is a micro-level analysis which, as we believe, can be integrated into more general methods that have been proposed by memory scholars to provide expert reports that are as immune as possible to bias (e.g., Otgaar et al., 2017; Vredeveldt et al., 2022; Arbiyah et al., 2023).

Of course, this work requires the intervention of memory experts with extensive and precise knowledge of how memory works (see Magnussen and Melinder, 2012). If a legal expert does not have this knowledge, it would seem necessary to call upon a memory expert to assist on these critical issues of eyewitness testimony (see fuller arguments in Dodier, 2018; Dodier et al., 2023). However, non-memory experts may sometimes have to give their opinion on memory phenomena, either because memory experts do not exist in the legal system (e.g., this is the case of France’s legal system, see Dodier, 2018; Dodier et al., 2023), or because memory phenomena are not at the heart of a legal case. In this respect, we advocate the idea that precise evidence-based guidelines and tools should be constructed and designed for use by expert witnesses (forensic or clinical) who are not memory specialists. In other words, memory specialists should work to create tools that can be used and adapted to the level of expertise of psychologists who carry out forensic examinations.

Although it has been shown that false memories are a “linguistic convenience” (Bernstein et al., 2018, p. 161), that is, the different types of false memories do not correlate particularly well, due to different and specific underlying processes, we find it crucial to examine future research aimed at clearly defining the processual differences behind false memories, but also the overlapping processes and mechanisms. For example, source-monitoring is generally invoked to explain natural distortions of the DRM type, but also the misinformation effect. Precisely identifying the limits and overlaps would make it possible to refine the model of testimony validity that we are advocating. Also, field studies using corroborated versus uncorroborated testimonies (e.g., Geraerts et al., 2007) could provide some external validity to the model.

We hope that this article will continue to bring balance to the debate on recovered memories of trauma, and that it will provide valuable resources for expert witnesses who must give their opinion in court cases based on eyewitness testimony, whether or not they include such recovered memories. Indeed, we can foresee that our contribution could be applied to contexts broader than this mere issue. If the place we give to recovered memories allows us to propose a model of testimony validity, it appears that, as we have just developed, its practical relevance can be found in all contexts of assessment of the validity of testimonies, whether the memories are continuous or recovered.

## Author contributions

OD: Writing – original draft. KB: Writing – original draft. CS: Funding acquisition, Writing – review & editing.
